# Isolated Superior Oblique Muscle Swelling Causing Acute Vertical Strabismus in Graves' Disease

**DOI:** 10.1155/2020/8829655

**Published:** 2020-11-01

**Authors:** Keiichi Aomatsu, Shunji Kusaka

**Affiliations:** ^1^Department of Ophthalmology, Nara Hospital, Kindai University Faculty of Medicine, Nara, Japan; ^2^Department of Ophthalmology, Kindai University Faculty of Medicine, Osaka, Japan

## Abstract

**Purpose:**

To report a case of isolated superior oblique muscle swelling causing acute vertical strabismus in Graves' disease.

**Case:**

A 26-year-old woman with a 1-month history of misalignment of the right eye and diplopia was referred to us. Her visual acuity and intraocular pressures were normal in both eyes, but eye movement tests showed clear misalignment of her right eye. Antibody tests for myasthenia gravis were negative. However, blood tests revealed abnormal levels of thyroid-related factors, such as decreased thyroid-stimulating hormone, elevated free T3 and T4, and elevated anti-thyroid-stimulating hormone receptor antibody. We performed magnetic resonance imaging (MRI), which showed slight enlargement of the left superior oblique muscle. The patient was eventually diagnosed with Graves' disease with superior oblique muscle involvement and underwent a thyroidectomy. Three months postoperatively, her diplopia and abnormal eye movements had substantially resolved.

**Conclusion:**

Isolated superior oblique muscle involvement may be a presenting symptom of Graves' disease. It should be taken into consideration that, in the early stages of thyroid-associated ophthalmopathy (TAO) in adults, only the superior oblique muscle may be enlarged.

## 1. Introduction

Thyroid-associated ophthalmopathy (TAO) is an autoimmune disease often encountered in daily clinical practice that can be accompanied by unique ocular findings such as exophthalmos and eyelid abnormalities. It is not difficult to diagnose TAO if hyperthyroidism is known or exophthalmos is apparent. However, when there is no obvious exophthalmos and the patient develops acute vertical strabismus, it is difficult to distinguish it from cranial nerve palsy. There have been some reports of patients with TAO whose diagnosis was difficult due to eye movement disorders similar to superior oblique muscle paralysis [1, 2]. In TAO, the inferior rectus and medial rectus muscles are commonly involved, and the superior oblique muscle is rarely involved, but that complicates eye movement disorders [3, 4]. In this report, we diagnosed TAO, but it was difficult to distinguish it from acute strabismus due to superior oblique muscle paralysis, and it was atypical for TAO because the oblique muscle was the only affected muscle on magnetic resonance imaging (MRI). As far as we know, there have been few reports in which only the superior oblique muscle was involved in cases of hyperthyroidism.

## 2. Case Report

**Figure 1:**
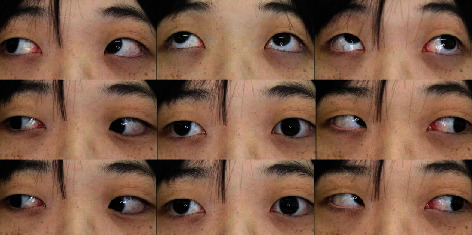
The patient's eye movement examination. The examination revealed overelevation of the right eye in adduction and restriction in the superior oblique muscle direction of the right eye, whereas movements of the left eye were almost normal.

**Figure 2:**
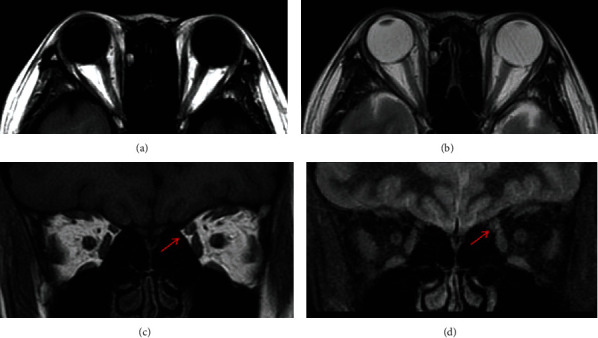
Magnetic resonance imaging (MRI) scans of the patient. The images showed a slight enlargement of the superior oblique muscle of the left eye (red arrow); however, neither enlargement nor abnormal signals were seen in the other four extraocular rectus muscles in either eye: (a) axial T1-weighted image, (b) axial T2-weighted image, (c) coronal T1-weighted image, and (d) coronal Short Tau Inversion Recovery (STIR) image.

A 26-year-old woman with a 1-month history of misalignment of the right eye and diplopia was referred to the Department of Ophthalmology, Kindai University Faculty of Medicine. Starting 2 years and 4 months prior to her referral to our clinic, she underwent local steroid injections for approximately 1 year, in the Department of Dermatology for alopecia areata. Her decimal visual acuity was 1.2 in each eye. Her intraocular pressure was 19 mmHg in the right eye and 20 mmHg in the left. The anterior ocular segment examination showed hypertropia of the right eye, but no conjunctival hyperemia or eyelid swelling was observed. Measurement of proptosis with a Hertel exophthalmometer showed 16 mm in each eye. Fundus examination showed no abnormalities. The misalignment of the right eye measured by an alternate prism cover test was 25-prism diopter (PD) hypertropia and 40 PD exotropia in the primary gaze position with fixation on a distant target. Eye movement examination revealed overelevation of the right eye in adduction and restriction in the superior oblique muscle direction of the right eye, with almost normal movements in the left eye (Figure 1). A dominant-eye test using a telescope showed that her left eye was dominant. In the first step of the Bielschowsky head-tilt test (BHTT), hypertropia of the right eye was observed. The second step of BHTT revealed an increase in right eye hypertropia with left gaze. The third step of BHTT was positive on right head tilts. The results of the BHTT measurements of vertical deviation were 35 PD and 14 PD on head tilts to the right and left, respectively. MRI showed a slight enlargement of the superior oblique muscle of the left eye, but neither enlargement nor abnormal signal of the four extraocular rectus muscles was seen in either eye (Figure 2). Since the patient was also aware of ptosis of the right eye, an antibody test for myasthenia gravis was performed. However, both antiacetylcholine receptor and anti-muscle-specific kinase antibodies were negative, and the Tensilon test was also negative.

A blood test, performed in the dermatology department three months earlier, showed abnormal thyroid-related values as follows. Thyroid studies revealed a decreased thyroid-stimulating hormone level of < 0.01 (normal, 0.5–5 *μ*IU/mL), an elevated free T3 level of 16.9 (normal, 2.3–4 pg/mL), an elevated free T4 level of 4.8 (normal, 0.9–1.7 ng/dL), and an elevated anti-thyroid-stimulating hormone receptor antibody level of 9.2 (normal, 0–2 U/L). From these findings, the patient was diagnosed with ophthalmopathy associated with Graves' disease, with superior oblique muscle involvement.

**Figure 3:**
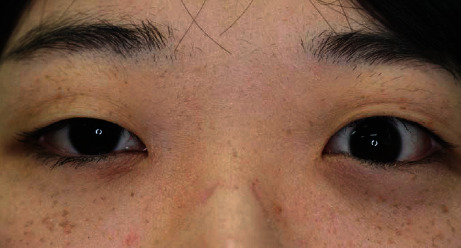
Clinical photograph of the patient at her last visit. Her eye position had improved.

For detailed investigation and treatment of Graves' disease, the patient was also referred to an endocrinologist and metabolic physician. Thyrotoxicosis, such as increased sweating and diffuse thyromegaly, was confirmed, and a high uptake rate was confirmed by a thyroid isotope test. Oral treatment with thiamazole was started, but it was changed to potassium iodide due to side effects such as fever and joint pain; no remission was achieved. Therefore, a total thyroidectomy was performed by her otorhinolaryngologist, about 3 months after her first visit to our clinic. Within a few weeks after the operation, her subjective symptoms, such as hand tremors and increased sweating, improved and diplopia gradually decreased. Four months after the operation, the patient transferred from our hospital to a hospital outside the prefecture, because of relocation, but diplopia and abnormal eye position had improved at the final visit (Figure 3).

## 3. Discussion

**Figure 4:**
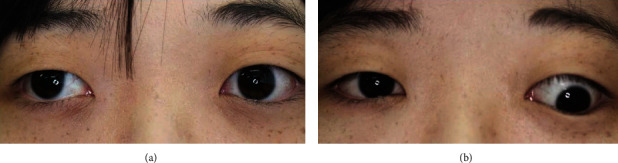
Clinical photographs of the patient. Eye positions are shown for (a) left-eye fixed and (b) right-eye fixed conditions. The original abnormality of the eye position was confirmed as shown in (b).

TAO is relatively often identified based on characteristic eyelid findings such as eyelid edema, eyelid retraction, and lid lag or exophthalmos. However, this case differed from typical TAO in that it caused acute-onset vertical strabismus due to isolated superior oblique muscle enlargement. TAO should have been raised to the differential diagnosis at an early stage, but there were some other points that made it difficult to reach the final diagnosis. One of the difficulties in this case was determining whether the abnormal eye position was due to a neurological disease or a muscle disease. At first, the patient's chief complaint was the right eye deviation, as shown in Figure 1, so we regarded this as ocular motor dysfunction in the right eye and suspected superior oblique palsy (consistent with the BHTT results). However, based on the findings on MRI and the patient's hyperthyroidism, we forced her to fixate with her right eye. When we did so, the original abnormality of the eye position was confirmed, as shown in Figure 4(b). Because the affected eye was the dominant eye, it was suspected that the fixation effort to correct the abnormal position of the left eye induced upward and outward deviation of the right eye. There have been some reports that patients with thyroid eye disease sometimes exhibit eye movement disorders such as superior oblique palsy [1, 2]. Additionally, Chen and Dagi and Kushner pointed out that BHTT may be positive in Graves' disease which may be erroneously diagnosed as superior oblique muscle paralysis [2, 5]. In our case, no neurological abnormalities other than acute strabismus were observed, and enlargement of the left superior oblique muscle was revealed on MRI. Therefore, we diagnosed the symptoms as due to a muscle abnormality rather than nerve palsy.

Gerlach and Ferbert proposed that endocrine ophthalmology could be divided into two types: those that cause exophthalmos, due to inflammation of the orbital adipose tissues, and those that cause diplopia and extraocular muscle paralysis due to inflammation of the extraocular muscles—the latter type is quite infrequent [6]. This case is also considered to be of the latter type, but such a pathological condition is rare, and caution should be taken in the diagnosis.

The other difficulty in this case was how to differentiate the cause of the hypertrophy of the isolated superior oblique muscle. Extraocular muscle enlargement and increased orbital fat are important findings of TAO, and such MRI findings are useful for diagnosis. Usually, multiple extraocular muscles are enlarged in one or both eyes, and various ocular misalignments are often seen in TAO. According to previous reports, the extraocular muscle that is most commonly involved in TAO is the inferior rectus muscle, followed by the medial rectus muscle, the superior rectus muscle, and the lateral rectus muscle, in that order [7]. There was a relatively rare case of TAO, with enlargement of the isolated lateral rectus muscle, reported [8], but reports regarding the superior oblique muscle are quite rare. There seems to have been only one article, in French, that showed hypertrophy of the isolated superior oblique muscle associated with Graves' disease on computed tomography [9], but this case is the first report to be demonstrated by MRI.

In this case, extraocular myositis and acquired Brown syndrome [10] also had to be considered differential diseases. Regarding myositis, this patient was considered negative because she did not complain of pain in the orbit, there was no eye injection due to inflammation, and movement of her left eye was not restricted. Brown syndrome was also considered negative because she had no history of head trauma or surgery, and MRI revealed swelling of the muscle belly rather than the trochlear or superior oblique tendon.

In conclusion, isolated superior oblique muscle involvement may be a presenting symptom of Graves' disease. It should be taken into consideration that, in the early stages of TAO in adults, only the superior oblique muscle may be enlarged.
